# Computerized working memory training for hypertensive individuals with executive function impairment: a randomized clinical trial

**DOI:** 10.3389/fnins.2023.1185768

**Published:** 2023-07-07

**Authors:** Regina Silva Paradela, Brenno Cabella, Mariana Penteado Nucci, Naomi Vidal Ferreira, Laura Aló Torres, Luiza Menoni Martino, Fernanda Marciano Consolim-Colombo, Luiz Aparecido Bortolotto, Danielle Irigoyen da Costa, Maria Claudia Irigoyen

**Affiliations:** ^1^Instituto do Coracao (InCor), Hospital das Clinicas HCFMUSP, Faculdade de Medicina, Universidade de São Paulo, São Paulo, Brazil; ^2^Institute of Theoretical Physics, São Paulo State University (IFT-UNESP), São Paulo, Brazil; ^3^Laboratory of Medical Investigations on Magnetic Resonance Imaging (LIM-44), Hospital das Clinicas HCFMUSP, Faculdade de Medicina, Universidade de São Paulo, São Paulo, Brazil; ^4^Division of Geriatrics, University of São Paulo Medical School, São Paulo, Brazil; ^5^Research Affairs Office, Amazonia Adventist College, Benevides, Pará, Brazil; ^6^Brain Institute (InsCer), Pontifícia Universidade Católica do Rio Grande do Sul (PUCRS), Porto Alegre, Brazil

**Keywords:** executive function, cognitive dysfunction, rehabilitation, hypertension, magnetic resonance imaging

## Abstract

**Background:**

Hypertension is associated with working memory (WM) impairment. However, the benefits of Cogmed WM training for the hypertensive population are unknown. Therefore, we aimed to evaluate Cogmed’s effects on the WM performance of hypertensive individuals with executive function (EF) impairment.

**Methods:**

We included 40 hypertensive patients (aged 40–70 years, 68% female) with EF impairment. They were randomized in a 1:1 ratio to receive 10 weeks of adaptive Cogmed training or a non-adaptive control training based on online games. The primary outcome was the WM performance. The secondary outcomes were verbal memory, visuospatial ability, executive function, global cognition, and the neuronal activity measured using functional magnetic resonance imaging (fMRI) under two WM task conditions: low (memorization of 4 spatial locations) and high (memorization of 6 spatial locations). An intention-to-treat (ITT) and per-protocol (PP) analysis were performed.

**Results:**

Cogmed did not show a significant effect on WM or any other cognitive outcome post-training. However, under the WM-low load and WM-high load conditions of the fMRI, respectively, the Cogmed group had an activation decrease in the right superior parietal lobe (ITT and PP analyses) and left inferior frontal lobe (PP analysis) in comparison to the control group.

**Conclusion:**

The Cogmed showed no effects on the WM performance of hypertensive individuals with EF impairment. However, activation decreases were observed in frontoparietal areas related to the WM network, suggesting a more efficient neuronal activity after training.

## Introduction

The worldwide population is aging fast, and the biggest concern of adults above 65 years or older is losing their memory and cognitive independence (Bassett and Folstein, 1993). Therefore, understanding strategies that could be helpful to improve adult cognitive functioning is of great clinical and public health interest.

Hypertension (HTN) is a well-known risk factor for cognitive impairment and dementia (Iadecola et al., 2016; Livingston et al., 2020; Canavan and O’Donnell, 2022). Studies have shown that HTN is associated with processing speed, attention, episodic memory, and executive function (EF) impairment. However, it is most related to EF dysfunction, including working memory (WM; Elias et al., 2012; Iadecola et al., 2016; Sánchez-Nieto et al., 2021; Eastman et al., 2022). Waldstein et al. comparing two groups of young men (23–40 years), found that the group with HTN had a poorer WM performance than the normotensive participants (Waldstein et al., 1996). Similarly, Shields et al. found that HTN was also associated with attention and WM deficits in women, based on their study involving 195 participants aged 45–55 years old from the New England Family Study (Shields et al., 2023). Furthermore, a population-based study that included 1,656 participants between 21 and 80 years of age showed that hypertensive patients, regardless of gender, had worse WM performance than normotensive individuals (Cansino et al., 2022). Functional near-infrared spectroscopy combined with cognitive testing also revealed that HTN was associated with WM impairment when compared to individuals with normal blood pressure values (Grant et al., 2015).

WM is an essential component of EF and is crucial for problem-solving and learning. It can be defined as the ability to store and manipulate information temporarily while we use it mentally (Baddeley, 1992). Given its presumed modifiability through repeated exposure to WM tasks, numerous computerized WM training programs have been developed for cognitive enhancement (Klingberg et al., 2005; Klingberg, 2010). These interventions have gained commercial popularity as potential non-pharmacological alternatives. Among them, Cogmed is a computerized training program designed to improve WM performance and has undergone extensive testing across various populations (Klingberg et al., 2002b; Løhaugen et al., 2011; Brehmer et al., 2012; Chacko et al., 2014; Anderson et al., 2018; Etherton et al., 2019; Flak et al., 2019). Some studies have reported benefits of Cogmed in enhancing WM performance (Løhaugen et al., 2011; Brehmer et al., 2012; Björkdahl et al., 2013; Chacko et al., 2014), but others did not (Flak et al., 2019). The evidence remains particularly limited and controversial regarding adults, with fewer studies conducted (Brehmer et al., 2012; Hyer et al., 2016; Simon et al., 2018; Flak et al., 2019; Khemiri et al., 2019; Henshaw et al., 2022). Some of them have indicated a beneficial impact of Cogmed on the WM performance of adults and older adults, with increased gains observed mainly in near-transfer tasks (Brehmer et al., 2012; Hyer et al., 2016; Simon et al., 2018; Khemiri et al., 2019; Henshaw et al., 2022). Conversely, other studies have found no differences in WM or other cognitive functions compared to the control group (Flak et al., 2019). However, as far as we know, no prior evidence exists regarding the effects of Cogmed on the WM performance of hypertensive adults.

Furthermore, studies have also examined the effects of Cogmed on neuronal activity (Klingberg et al., 2002a; Brehmer et al., 2011). In a sample of 23 young adults, Brehmer et al. observed a decrease in neocortical brain activity in the Cogmed group, suggesting increased neural efficiency following training (Brehmer et al., 2011). Other studies have reported similar changes in post-training neuronal activation in adults (Emery et al., 2008; Belleville et al., 2014). For individuals with HTN, there is currently a lack of studies investigating the effects of Cogmed training (or similar interventions) on brain function using functional magnetic resonance imaging (fMRI). However, previous literature has demonstrated that learning specific tasks or experiences can lead to structural changes in the brain (Maguire et al., 2000; Draganski et al., 2004; Mechelli et al., 2004). Additionally, studies utilizing blood-oxygen-level-dependent (BOLD) methods have shown alterations in brain function due to such learning processes (Klingberg et al., 2002b; Brehmer et al., 2011). Hence, the objective of our study was to evaluate the effect of Cogmed training on WM performance in hypertensive individuals with EF impairment. Our secondary outcomes included EF, verbal memory, visuospatial ability, global cognition, as well as the indirect measurement of neuronal activity using fMRI.

## Methods

### Data availability statement

All the data and materials used in this study are available or accessible on request.

### Study design

This randomized clinical trial (RCT) study was approved by the local ethics committee of the Heart Institute of the University of São Paulo (Approval registration number: SDC 4266/15/093). All participants gave written informed consent, and the study was registered at ClinicalTrials.gov (NCT02738034).

### Participants

We included hypertensive patients from the Heart Institute of the University of São Paulo Medical School. Inclusion criteria were age between 40 and 70 years old, a previous diagnosis of HTN (according to a medical diagnosis recorded in the hospital’s database or a report of antihypertensive drug use), access to a computer with internet at home, no previous experiences with cognitive training, no clinical limitations to hearing or seeing properly, and EF impairment. Exclusion criteria were less than 4 years of education, severe cognitive or communication impairments, history of stroke, secondary HTN, head trauma, substance abuse, and contraindications to performing an fMRI. Sociodemographic and clinical variables were self-reported in the baseline interview.

We evaluated the baseline EF impairment using the Digit Span Forward (DSF) and Backward (DSB) subtests from the Wechsler Memory Scale-Revised (WMS-R), and the FAS letters fluency test from the Controlled Oral Word Association Test (COWAT). A Z-score was calculated for each of these tests by subtracting the participant’s test score from the mean of a comparative sample score and dividing the difference by the standard deviation (SD) of this sample. These comparative values were extracted from normative tables providing scores from healthy populations matched by age and education (Wechsler, 1987; Tombaugh et al., 1999). Thus, a *Z*-score equal to or greater than −1 in at least one of these tests was considered an EF impairment.

### Adaptive training

We used a computerized and standardized adaptive WM training program (Cogmed®) commercially available from Pearson Education as the intervention method.

The training program was composed of 30 sessions. In the first session, the participants started each task at the same difficulty level (i.e., low difficulty). As the training continued, the program automatically adjusted the level according to the participant’s performance. Each training session started at the task difficulty level where the participant ended the previous session at. Participants could not train more than once a day (after completing a session, the program did not allow new access on the same day) and were instructed to train 3 days a week for 10 weeks to achieve the 30 sessions.

Participants did the training at home using their personal computers. They worked on five of ten visuospatial and verbal WM tasks that were made available automatically by the program in each session, totaling an average of 35 min per day. Before starting, subjects received training instructions and were advised to find a peaceful time and place to train. The program continuously recorded their performance and information regarding the time they spent on each session. They were contacted via telephone weekly to receive motivation and feedback on their training process.

### Control group

The control group also made a computerized training of 30 sessions based on four simple online games: hangman, tic-tac-toe, nim game, and naval battle (Supplementary Table 1). The same four games were programmed to be available every session for 8 min and 75 s each, so they took an average of 35 min of training per session.

We developed a webpage to put the games together and manage all information regarding the control training. The control group accessed this website through a login and password similar to the Cogmed training. Participants were instructed to train three sessions per week, but not on the same day. Once they finished the training, access to the games was programmed to be available again only on the next day. In this way, they could also complete the 30 sessions in about 10 weeks of training. They also received telephone calls once a week. But compared to Cogmed training, the control group’s activity was simpler and was not designed to increase the performance of any specific cognitive function (Subramaniam et al., 2014).

### Cognitive outcomes

The participants were assessed at two-time points (baseline and post-training) using the DSF and DSB subtests from the WMS-R, Letter-Number Sequencing (LNS) from the Wechsler Adult Intelligence Scale-Third Edition (WAIS-III), copy and delayed recall of Rey Complex Figure test (RCF) (at the baseline) and Taylor Figure (at the post-training evaluation), immediate and delayed recall of Stories A and B from the WMS-R (at the baseline and post-training evaluation, respectively), FAS letter fluency from the COWAT, Frontal Assessment Battery (FAB), and the Mini-Mental State Examination (MMSE). We used different available versions of tests to assess the visuospatial abilities and verbal memory performance at baseline and post-training evaluations to minimize the learning and re-test effect. A detailed description of all tests was given before (Paradela et al., 2021).

A *Z*-score was calculated for each test by subtracting the participant’s test score from the mean sample score and dividing the difference by the sample SD. Thus, a *Z*-score of −1 represents a cognitive performance of 1 SD below the mean sample score for each test (Rawlings et al., 2014; Paradela et al., 2021). A composite WM *Z*-score was calculated by averaging the *Z*-scores of the DSB subtest from WMS-R and LNS from WAIS-III. A composite verbal memory *Z*-score was calculated by averaging the Z-scores of the two parts of the stories’ subtests from the WMS-R and then standardizing this mean (Rawlings et al., 2014; Paradela et al., 2021). The same was made for the RCF and Taylor Figure to obtain a composite *Z*-score for visuospatial ability. The DSF subtest from WMS-R, the FAS letters fluency test, and the FAB were taken together in a composite EF *Z*-score. The MMSE Z-score was considered alone as a measure of global cognition. Thus, the primary outcome of this study was the composite *Z*-score of WM. The secondary outcomes were EF, verbal memory, visuospatial ability, global cognition composite *Z*-scores, and neuronal activation measured by fMRI (described below). To assess the EF outcome, we calculated the z-score considering tests that measure other components of EF, except WM, which was considered a separate outcome.

### Functional magnetic resonance imaging

Participants were scanned (pre and post-training) using a 3.0 T MR system (Philips Achieva, Eindhoven, the Netherlands) that was equipped with 80 mT/m gradients and a 32-channel head coil. The parameters for the functional image acquisition are described in the supplementary section. Morphometric image acquisition was previously described (Paradela et al., 2021).

During the fMRI exam, participants performed a WM factorial block-design task adapted from Brehmer et al. under two WM load conditions (low vs. high) and their respective controls (Klingberg et al., 2002a; Brehmer et al., 2011). A block-design task was used, with randomized order of WM-low, WM-high, and the two control conditions (Klingberg et al., 2002a; Brehmer et al., 2011). Stimuli were programmed and presented using E-prime (Version 1.2), which also recorded behavioral performance (accuracy and reaction time).

#### WM task conditions

**Figure 1:**
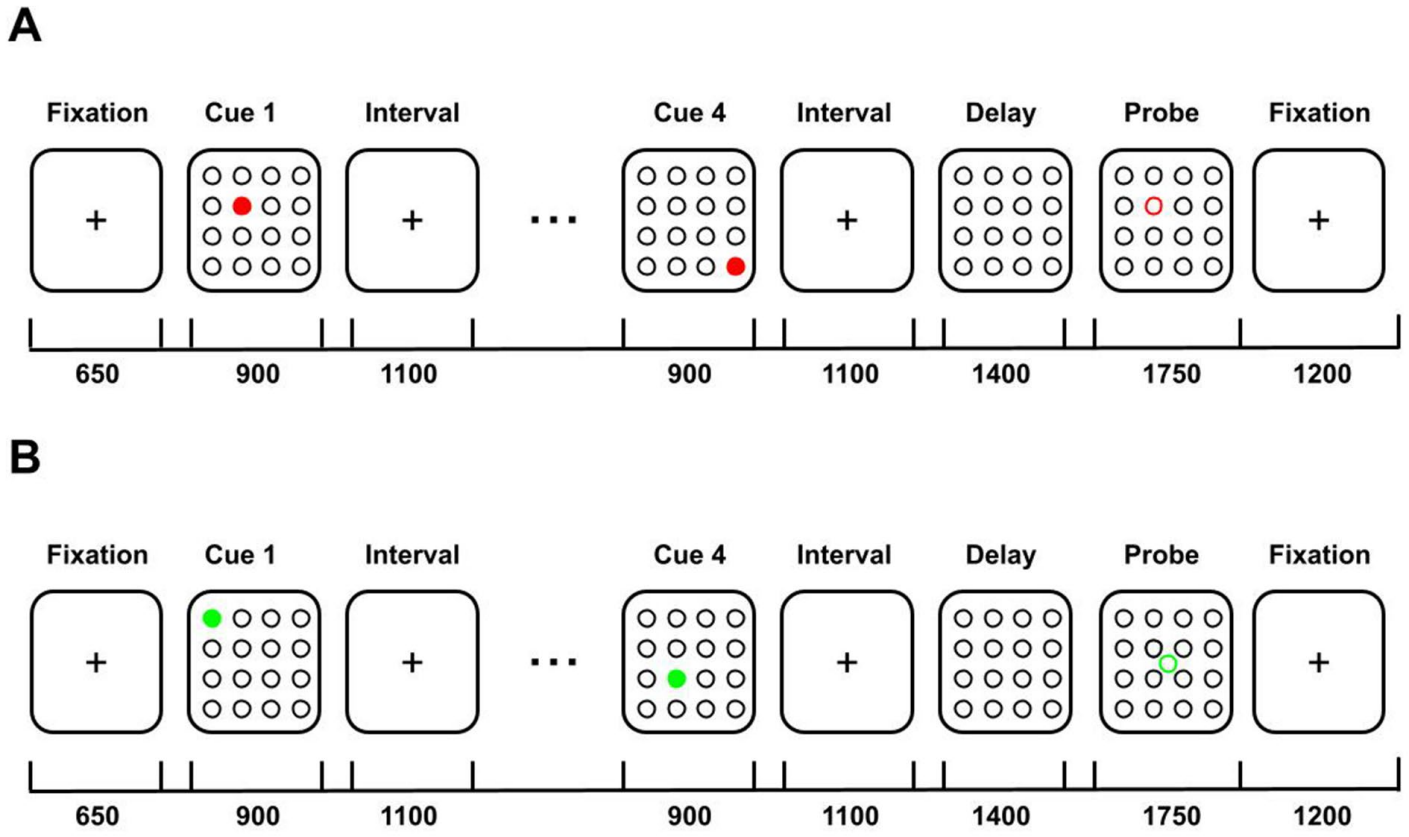
Illustration of the WM-low load task and their control condition performed during the functional magnetic resonance imaging exam. **(A)** WM-low load task cues were presented in a 4 × 4 grid. After a delay, individuals had to indicate if a probe was in the same location that any of the previous cues appeared. **(B)** Control condition. Individuals were oriented to just look at the image but not memorize them. The numbers below the line represent the display time in ms. Image adapted from Brehmer et al. (2011).

On display, the individuals saw either 4 (WM-low load) or 6 (WM-high load) red-filled circles appearing sequentially in a 4 × 4 grid. After a 650 ms delay, each cue was presented for 900 ms, followed by an interval of 1,100 ms (WM-low load) or 500 ms (WM-high load). Participants were asked to remember the locations in which the red-filled circles appeared. After another delay of 1,400 ms (WM-low load) or 1,000 ms (WM-high load), a response phase of 1750 ms followed. In the response phase, an unfilled probe circle was presented on the 4 × 4 grid, and participants had to indicate whether the probe was in the same location where any previous cues appeared. Responses were acquired by pressing a button with the right index finger to indicate “yes” or the right middle finger to indicate “no.” The next trial started after a 1,200 ms delay (Figure 1; Brehmer et al., 2011).

#### Control conditions

For the control conditions, individuals were exposed to 4 (Control-low) or 6 (Control-high) green-filled circles that were also presented sequentially in a 4 × 4 grid. Each image had an exposition time similar to the WM tasks. However, they were oriented for not memorizing the sequences. In the response phase, an unfilled green circle appeared in the center of the grid, and participants had to press any button when the probe appeared (Brehmer et al., 2011).

Participants performed five blocks in each condition (WM-low, WM-high, Control-low, and Control-high), alternated in a counterbalanced order, and split across two runs of 6.000 ms. Each block contained three trials, yielding a total of 15 trials per condition.

#### Neuroimaging analysis

We used the FSL version 6.0^1^ to analyze the fMRI data. The brain volumes were processed for brain mask extraction (BET), movement correction (MCFLIRT), spatial smoothing (FWHM = 5 mm), and spatial normalization to standard space (in a two-stage process: EPI low-resolution images registration to the same subject’s T1-weighted structural images using the Boundary-Based Registration (BBR) cost function, and the resulting transformation registration to MNI-152 standard space using 12 DoF). The activation maps were created using a two-predictor general linear model (GLM) developed in FILM (WM-low and WM-high load conditions). The standard hemodynamic response function was convolved with a double gamma function to model all predictors. We considered in the first-level analysis the individual maps of the contrasts WM-low>Control-low, WM-high>Control-high, WM-low>WM-high, and WM-high>WM-low. In the group maps (high-level analysis), the differences between groups (Cogmed vs. control) over time (before and after training) were identified using the mixed effect model (FLAME 1) and clusterwise inference. The Z (Gaussianised T/F) statistic images were non-parametrically thresholded based on clusters defined by a Z-score greater than 2.3, and a corrected cluster significance threshold of *p* = 0.05 (Worsley, 2001). Moreover, we extracted the beta values of the BOLD signal from the activation cluster corresponding to the WM-low (Cogmed vs. control) and WM-high (Cogmed vs. control) contrasts for each subject at the post-training. This extraction was performed using the Featquery processing routine provided by FSL.

### Sample size calculation

Since there was no prior study evaluating the cognitive performance of hypertensive individuals post-Cogmed training, sample size and power calculation were done by using scores pre and post-Cogmed training (mean and SD) from the DSB subtest from the WMS - R obtained by Brehmer et al. (2012). We determined that 32 participants were needed for inclusion in the study to obtain a statistical power of 80% at an alpha level of 0.05 for differences in the WM subtest. Considering the dropout possibilities, we randomized a total of 40 participants (20 per group).

### Randomization

Participants were randomly assigned in a 1:1 ratio to the adaptive group that did the Cogmed training or to a non-adaptive control group that did training based on online games. The random allocation sequence was computer-generated and kept by a person without involvement with the study. Then, the first author (RSP), who enrolled and assigned participants to the interventions, was blinded for the allocation list before the assignment. The trained psychologists (LMM and LAT) that performed the cognitive evaluation were blind to the group allocation before and after the assignment.

### Clinical evaluation

Systolic blood pressure (SBP), diastolic blood pressure (DBP), and heart rate (HR) were measured on the baseline interview using an Omron automatic device (model HEM-705 CPINT) in the right upper arm, with the subject seated, after 5 min of resting according to the VII Brazilian Guideline on HTN (Sociedade Brasileira de Cardiologia et al., 2010). A mean of 2 measurements with a 1-min interval was calculated and used to determine each patient’s SBP, DBP, and HR.

### Statistical analysis

We presented the data as the mean and standard deviation (SD) for normally distributed variables and the median and interquartile range (IQR) for non-normally distributed variables. Categorical variables were described as relative frequencies. For comparisons between the groups, we used Student’s *t*-test for normally distributed continuous variables, the Mann–Whitney test for non-normally distributed continuous variables, and the Chi-squared test or Fisher exact test for categorical variables.

We investigate the effect of the training (Cogmed vs. control) and the time (pre and post-training) using a linear mixed model for repeated measurements with a random intercept for each subject and maximum likelihood estimation. The outcome variables were the composite Z-scores of WM, verbal memory, visuospatial ability, EF, and global cognition in models adjusted for age, sex, and education. In addition, we also verified the effect of the training and the time on the accuracy (number of correct answers) and reaction time (in ms) of the WM tasks performed during the fMRI exam.

We performed an intention-to-treat analysis (ITT), including all randomized participants that completed the follow-up (post-training evaluation) even if they had not completed or started the training. In addition, we made a per-protocol analysis (PP) with those who completed at least 20 sessions of the training. The ITT analysis was conducted to preserve the number of subjects indicated by the sample size calculation and to avoid potential bias due to the exclusion of some patients. The PP analysis mimics the optimal conditions to evaluate the training effects once it included only participants who completed at least 80% of the training (20 of 30 sessions). Cohen’s d-effect size analysis was performed to measure the magnitude of the differences in cognitive outcomes between the two groups.

Furthermore, we analyzed the correlation of the beta values derived from the post-training activation of WM-Low and WM-high contrast maps with the post-training Z-score of WM among individuals in both the Cogmed and control groups. Pearson’s correlation coefficient (r) was employed for this purpose.

All analyses were performed using R-4.0.0, and we considered an alpha level of 5% after the Bonferroni adjustment for multiple comparisons.

## Results

**Figure 2:**
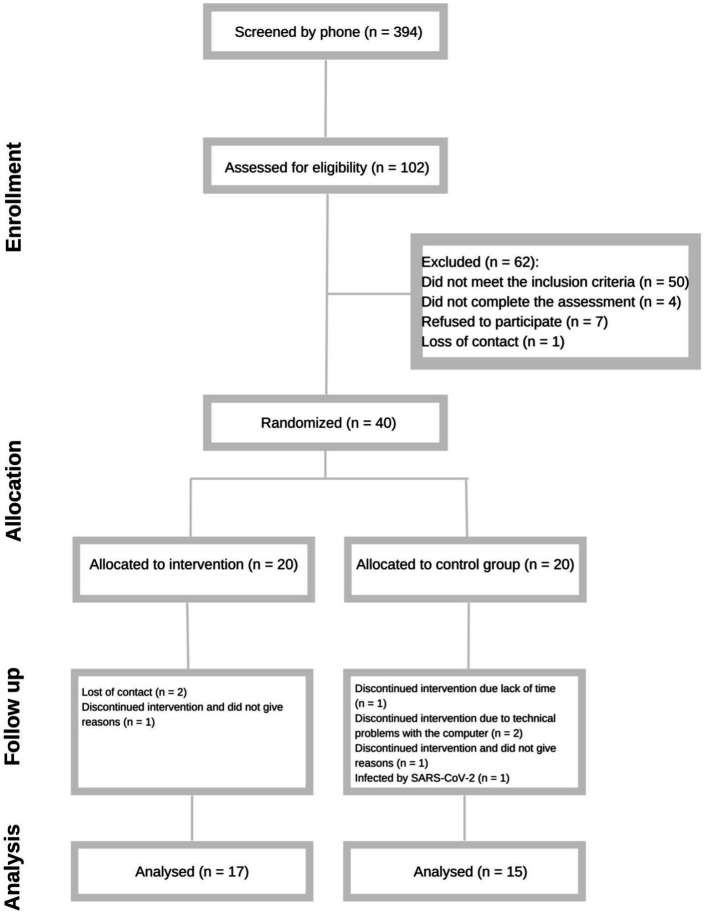
Enrollment of the participants.

We screened 394 possible eligible participants by phone from September 2016 to December 2019. Of 102 participants that agreed to participate and were evaluated at baseline, we had 40 individuals that met all inclusion criteria. Eight subjects withdrew from the study: three from Cogmed and five from the control group. The main reason in both groups was lack of time to realize the training (*n* = 1), loss of contact (*n* = 2), withdrawal without giving reasons (*n* = 2), technical problems with the computer (*n* = 2), and an impediment to performing the training because of an infection by the SARS-CoV-2 virus during the Covid-19 pandemic (*n* = 1). Thus, we included in the ITT analysis 17 participants from the Cogmed group and 15 participants from the control group (Figure 2). In the PP analysis, we included 13 participants from the Cogmed group and 11 participants from the control.

### Baseline characterization of the sample

The groups were similar in age, sex, race, education, and family monthly income (Table 1). However, in the PP analysis was a borderline difference in age (*p* = 0.05). The median age of the Cogmed group was 58 years (IQR = 47–65 years) compared to the median age of 65 years (IQR = 56–69 years) in the control group. In addition, the control group had a higher frequency of never smoked participants than the Cogmed group in both ITT (*p* = 0.03) and PP (*p* = 0.01) analysis (Table 1).

**Table 1:** Baseline characteristics of the sample.

	ITT analysis			PP analysis		
Variables	Control (*n* = 15)	Cogmed (*n* = 17)	*P*	Control (*n* = 11)	Cogmed (*n* = 13)	*P*
**Age (years), median (IQR)** ^a^	63 (58–68)	58 (47–61)	0.17	65 (56–69)	58 (47–65)	0.05
**Sex (female), %** ^c^	76.5	73.3	1.00	76.9	72.7	1.00
**Race, %** ^d^			0.21			0.52
White	35.3	60		46.1	63.6	
Black	23.5	20		7.7	9.1	
Brown	41.2	13.3		46.1	18.2	
Other	0	6.7		0	9.1	
**Education (years), median (IQR)** ^a^	12 (12–13)	12 (7–16)	0.78	12 (12–13)	12 (7–16)	0.88
**Estimated IQ, mean ± SD** ^b^	95 ± 7	96 ± 8	0.88	96 ± 7	98 ± 7	0.50
Monthly income*^a^, median (IQR)	5 (5–7)	5 (2–6)	0.20	5 (5–13)	3 (2–16)	0.09
**Diabetes, %** ^c^	5.9	13.3	0.91	7.7	18.2	0.58
**Dyslipidemia, %** ^c^	29.4	60	0.17	23.1	63.6	0.11
**Smoking, %** ^c^			0.03			0.01
Never	88.2	46.7		100	54.5	
Current	0	13.3		0	0	
Past	11.8	40		0	45.5	
**Alcohol use, %** ^c^			0.51			0.32
Never	29.4	20		38.5	18.2	
Current	71	66.7		61.5	63.6	
Past	0	13.3		0	18.2	
**BMI (Kg/m**^2^**), median (IQR)** ^a^	31 (30–33)	30 (24–34)	0.79	31 (27–32)	27 (24–34)	0.78
**SBP (mmHg), mean ± SD** ^b^	151 ± 29	141 ± 22	0.28	149 ± 25	149 ± 25	0.85
**DBP (mmHg), mean ± SD** ^b^	90 ± 16	86 ± 13	0.51	86 ± 11	90 ± 13.5	0.48
**Heart rate (bpm), mean ± SD** ^b^	69 ± 10	74 ± 9.5	0.14	67 ± 11	74 ± 9	0.10
**Time of hypertension (years), mean ± SD** ^b^	19.9 ± 9.3	17.2 ± 13.3	0.51	18.8 ± 10	17.7 ± 14.2	0.83
**Number of medications, mean ± SD** ^b^	5 ± 2	4 ± 1.5	0.34	5 ± 2	4 ± 2	0.32
**Most frequently used medications, %** ^c^						
ARB	64.7	40	0.30	53.8	45.5	1.00
ACE inibitors	29.4	46.7	0.52	38.5	36.4	1.00
Diuretics	82.4	60	0.31	76.9	63.6	0.79
CCB	41.2	46.7	1.00	53.8	54.5	1.00
BB	47.1	53.3	1.00	46.2	45.5	1.00
Other	52.9	73.3	0.41	53.8	81.8	0.31
**Raw cognitive score, median (IQR)** ^a^						
DSF from WMS-R	6 (5–6)	5 (4–5)	0.07	6 (4.5–6)	5 (4–6)	0.37
DSB from WMS-R	4 (3.5–5)	4 (3.5–5)	0.38	4 (3.5–5)	4 (3.5–4)	0.30
LNS from WAIS III	7 (5.5–9.5)	6 (4–7)	0.05	7 (5.5–8.5)	5 (4–6)	0.06
Immediate recall of stories A from WMS-R	14 (11–18)	16 (13–17)	0.51	13 (11–16)	16 (13–17)	0.34
Delayed recall of stories A from WMS-R	9 (6.5–12)	13 (11–14)	0.03	9 (6.5–10)	13 (10–14)	0.04
Copy of RCF	33 (32.5–36)	32 (27.5–36)	0.27	33 (32–35)	32 (27.5–35)	0.16
Delayed recall of RCF	13 (11.5–17)	15 (11.5–16.5)	0.78	12 (10.5–15.5)	15.5 (13.5–16.5)	0.16
FAS letters fluency test	27 (23–31.5)	27 (25–29)	0.98	28 (24–34.5)	26 (24–30)	0.66
FAB	15 (14–16)	14 (12–15)	0.09	15 (14–16)	14 (11–15)	0.16
MMSE	28 (26–29)	26 (26–28)	0.16	28 (26.5–29)	27 (26–28)	0.41

ITT, intention to treat analysis. PP, per-protocol analysis. SBP, systolic blood pressure. DBP, diastolic blood pressure. BMI, body mass index. ACE, angiotensin-converting enzyme; ARB, angiotensin receptor blockers; BB, beta-blockers; CCB, calcium channel blockers.

a Mann–Whitney *U* test;

b Student’s *t*-test.

c Chi-square test.

d Fisher exact test.

*Number of Brazil’s national minimum wages.

The mean SBP was 141 ± 22 mm Hg in the Cogmed group and 151 ± 29 mm Hg for the control group. The mean DBP was 86 ± 13 mm Hg for the participants in the Cogmed group and 90 ± 16 mm Hg for the controls. There were no significant differences between the groups for SBP (*p* = 0.28) and DBP (*p* = 0.51; Table 1). The mean time of HTN since diagnosis was 17.2 ± 13.3 years in the Cogmed group and 19.9 ± 9.3 years in the control group, and no differences were found between them (*p* = 0.51). There were also no differences in the HR frequency, number of medications, and the most frequently used antihypertensive drugs. Similar results were found in the PP analysis (Table 1).

At the baseline, the groups had similar raw scores in all cognitive tests, except for Delayed recall of stories A from WMS-R, since the Cogmed group had a better performance than the control in ITT (*p* = 0.03) and PP (*p* = 0.04) analysis (Table 1).

### Cognitive outcomes

Neuropsychological evaluation was performed on average 3 ± 1.5 months post-training for the participants included in the ITT analysis (*n* = 32). For the individuals included in the PP analysis (*n* = 24), the cognitive evaluation was realized on average 1.2 ± 1.4 months post-training.

There was no significant effect of the intervention and time on the composite Z-scores of the WM (primary outcome), verbal memory, EF, and global cognition in analysis adjusted for age, sex, and education (Table 2). We found a significant effect of the interaction between the groups and time on the visuospatial ability composite *Z*-score (*p* = 0.04). However, this difference did not remain significant in the *post hoc* analysis with Bonferroni correction. In the PP analysis, the groups and times did not differ in any cognitive domain (Table 2). Post-training measurement effect size calculated using Cohen’s d is presented in Table 2 for both ITT and PP analysis.

**Table 2:** Effect of intervention and time on cognitive outcomes.

	Control group (*n* = 15)		Cogmed group (*n* = 17)		*P*			Effect size measurement	
	Baseline	Post-training	Baseline	Post-training	Groups	Times	Groups*times	Cohen’s d effect size	Magnitude
Intention-to-treat analysis									
*Primary outcome*									
Working memory	0.31 ± 1.1	0.11 ± 1.1	−0.29 ± 0.82	−0.10 ± 0.97	0.07	0.72	0.12	0.21	Small
*Secondary outcomes*									
Executive function	0.25 ± 0.93	0.21 ± 1.1	−0.22 ± 1.0	−0.18 ± 0.92	0.15	0.71	0.70	0.39	Small
Verbal memory	−0.15 ± 1.3	−0.40 ± 1.0	0.13 ± 0.65	0.37 ± 0.84	0.13	0.92	0.21	−0.82	Large
Visuospatial ability	0.18 ± 0.75	−0.17 ± 1.0	−0.16 ± 1.2	0.15 ± 0.97	0.92	0.60	0.04	−0.32	Small
Global cognition	0.26 ± 0.94	0.09 ± 0.89	−0.23 ± 1.0	−0.08 ± 1.11	0.15	0.57	0.50	0.17	Insignificant
Per-protocol analysis	Control group (*n* = 11)		Cogmed group (*n* = 13)						
*Primary outcome*									
Working memory	0.26 ± 1.2	−0.10 ± 1.1	−0.42 ± 0.88	−0.22 ± 1.1	0.12	0.53	0.08	0.12	Insignificant
*Secondary outcomes*									
Executive function	0.27 ± 1.1	0.27 ± 1.2	−0.17 ± 1.2	−0.03 ± 0.98	0.20	0.86	0.61	0.29	Small
Verbal memory	−0.21 ± 1.4	−0.28 ± 1.0	0.12 ± 0.74	0.35 ± 0.92	0.30	0.67	0.56	−0.64	Moderate
Visuospatial ability	−0.01 ± 0.72	−0.24 ± 0.62	−0.03 ± 1.2	0.31 ± 1.02	0.23	0.94	0.12	−0.63	Moderate
Global cognition	0.30 ± 0.95	−0.17 ± 0.79	−0.04 ± 0.99	0.06 ± 1.2	0.49	0.18	0.19	−0.22	Small

*p* values from linear mixed model analysis for repeated measurements adjusted for age, sex, and education.

Cohen’s *d* effect size for differences between the groups in post-training measurement.

Mean ± SD of the composite *Z*-scores.

### Brain activation

**Figure 3:**
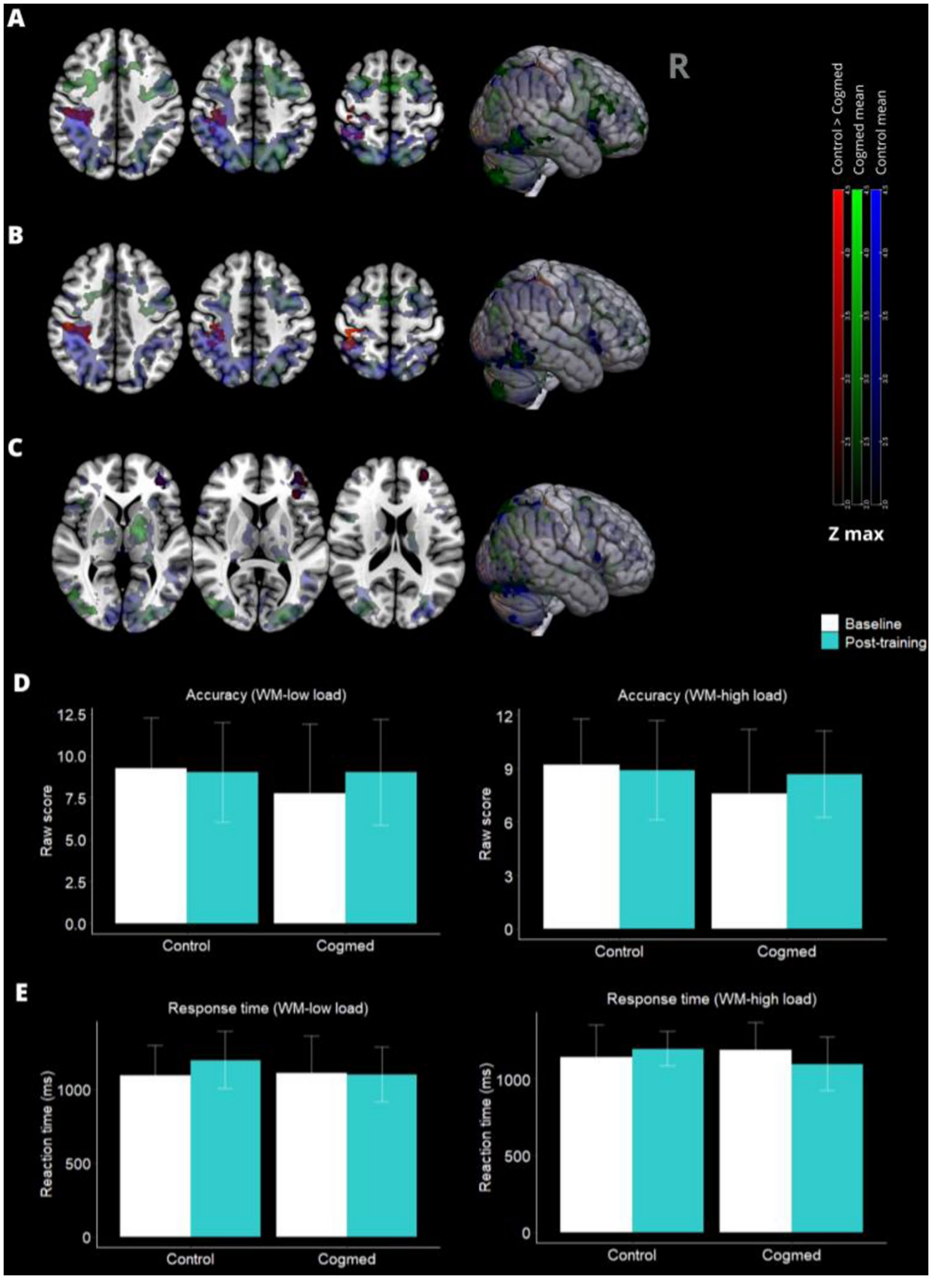
Post-training brain activity maps and behavioral performance. **(A)** In the right superior parietal lobe activation was higher in the control groups than in the Cogmed group after training during the working memory (WM) low-load task condition in the intention-to-treat analysis (ITT) (*p* < 0.05*). **(B)** The Same result was observed in per-protocol (PP) analysis. **(C)** Neuronal activation in the left anterior frontal lobe under the WM-high load condition was higher in the control group compared to the Cogmed group (*p* < 0.05*), but only in the PP analysis. There was no difference between the groups in behavioral performance (**D,E**: ITT analysis). *p-values for cluster-wise thresholded *z*-score equal to or greater than 2.3.

In the baseline activation map comparing the WM tasks and their control conditions across the groups, frontal–parietal-occipital WM network areas were activated as expected (Brehmer et al., 2011). There were no baseline differences in the load conditions between the two groups (data not presented). Following training, Cogmed had an activation decrease in the right superior parietal lobe compared to control under the WM-low load condition in both the ITT (Figure 3A; Table 3) and PP analysis (Figure 3B; Table 3). We also observed a decreased activation in the left anterior frontal lobe of the Cogmed group compared to the control under the WM-high load condition, but only in the PP analysis (Figure 3C; Table 3). The individual maps of contrasts between Low vs. High and High vs. Low showed that the Cogmed group had, on average, a post-training activation increase in the parietal and occipital lobe areas (Supplementary Figures 1, 2; Table 3).

**Table 3:** Post-training activation changes in the working memory (WM)-low and WM-high conditions.

Brain area	Voxels	*x*	*y*	*z*	*Z* max
Cogmed activations decrease*					
*WM-low – ITT analysis (n = 26)*					
Right superior parietal lobe	855	44	−44	60	3.57

*WM-low – PP analysis (n = 21)*					
Right superior parietal lobe	618	40	−40	58	3.56

*WM-high – PP analysis (n = 21)*					
Left anterior frontal lobe	344	−38	46	4	3.68
Cogmed activations increase†					
*WM-low > WM-high – ITT analysis (n = 26)*					
Right/Left Parietal lobe	409	2	−62	30	3.51

*WM-high > WM-low – ITT analysis (n = 26)*					
Right Occipital lobe	509	30	−86	4	3.37

Coordinates *x*, *y*, and *z* are reported in MNI space.

^*^Activations decreases compared to the control group.

^†^On average, the subjects in the Cogmed group had an increase in activation in these regions.

Although no significant difference was found in the composite Z-score of WM performance after Cogmed training, we observed an important correlation among participants in the Cogmed group who completed at least 20 training sessions (PP analysis). Specifically, we found a significant correlation between low values of activation in the right superior parietal lobe derived from the in-scanner WM-Low load task condition and a higher composite Z-score of WM post-training (*r* = −0.75, *p* = 0.007). However, no significant correlation was detected between the beta values of activation during the WM-Low load task condition and the WM performance evaluated outside the scanner in the Cogmed group included in the ITT analysis (*r* = −0.42, *p* = 0.12).

Additionally, no correlation was found between post-training activation in the left anterior frontal lobe derived from the WM-high load task condition and the Z-score of WM among individuals of the PP analysis in both the Cogmed and control groups. Furthermore, in the control group, there were no post-training significant correlations observed between the beta values of activation obtained during the WM-Low (ITT and PP analyses) and WM-High (PP analysis) conditions, and the WM performance evaluated outside the scanner (Supplementary Table 2).

Furthermore, the two groups did not differ in accuracy or response time for the in-scanner WM tasks performed during the fMRI assessment. Neither on the ITT (Figures 3D,E) nor PP analysis (not presented).

## Discussion

In this RCT in hypertensive individuals with EF impairment, the Cogmed training was not effective in enhancing the gains for WM, verbal memory, visuospatial ability, EF, and global cognition. However, we found evidence of decreased neuronal activity in the frontoparietal areas of the Cogmed group in comparison to the control. In addition, among the Cogmed participants included in the PP analysis, a higher composite Z-score of WM was correlated with lower beta values of activation in the right superior parietal lobe obtained during the WM-low load task performed inside the scanner.

Previous studies have found an association between adaptive Cogmed training and WM improvement (Hyer et al., 2016; Simon et al., 2018; Khemiri et al., 2019), but others have not (Flak et al., 2019). Flak et al., in a double-blind RCT, investigated the effect of a five-week version of the Cogmed training (with 20–25 sessions) in 68 adults (aged 43–88 years) with mild cognitive impairment (MCI). However, similar to our results, they found no differences between the Cogmed group and the non-adaptive training control group on the primary outcome of WM or in any of the secondary cognitive outcomes (attention, processing speed, visual learning, verbal memory, and EF; Flak et al., 2019). On the other hand, another study in 68 older adults (≥65 years old) with MCI evaluated the effectiveness of Cogmed (also the five-week version) in comparison to a non-adaptive control version in the cognitive performance (Trials B, LNS, and Span board tests). Both groups had improved over time in all cognitive measures, but the Cogmed group’s gains were superior only in a visuospatial WM test (the Span board test) that is very similar to the tasks trained by the Cogmed program (Hyer et al., 2016). Furthermore, 82 cognitively normal adults (mean age of 73 years), after 5 weeks of Cogmed training, were also assessed with EF tests (Trail Making Test Part A and Part B, Digit Symbol, COWAT, and Semantic Fluency). The Cogmed group, compared to the control, showed a significant improvement only in the Digit Symbol test (Simon et al., 2018). Likewise, an RCT in 50 patients with alcohol use disorder (50 years old at mean) found gains related to Cogmed, but only in the Digit Span test (Khemiri et al., 2019). Recently, Henshaw et al. showed results from an RCT that examined the gains related to 5 weeks of Cogmed training in 57 hearing aid users aged 50 to 74. They found improvements in the DSB test and a small improvement in self-reported hearing ability in the Cogmed group compared to the control (Henshaw et al., 2022). Summarizing, the benefits of Cogmed as an isolated approach for the cognitive rehabilitation of adults have been restricted and more evident in tests similar to the trained tasks (i.e., Digit Span tests). In addition, scarce evidence of the transfer effect for non-trained tasks has been found. Further investigations should test the combined effect of Cogmed with other strategies for the cognitive enhancement of hypertensive populations.

Regarding brain function, previous studies on adults have reported decreased activity in frontoparietal areas underlying WM after Cogmed training, in agreement with our findings (Brehmer et al., 2011; Vermeij et al., 2017). Brehmer et al. examined the neural activity of 23 healthy older adults (aged 60 to 70 years) before and after 5 weeks of the Cogmed training. Brain activity was measured using fMRI while subjects performed a WM task under two difficult conditions (WM-low and WM-high load conditions; Brehmer et al., 2011). They found that during the WM-high load condition, activity in cortical areas (dorsolateral prefrontal cortex, superior temporal, and lingual gyrus) decreased more in the adaptive training group than in control. Vermeij et al. also demonstrated a prefrontal activity decrease in healthy older adults (*n* = 21) at a high load verbal n-back task after 5 weeks of Cogmed training. However, the same was not found for patients with MCI (*n* = 14; Vermeij et al., 2017). Similarly, other studies have reported brain activity changes in WM cortical areas of adults after computerized WM training, supporting the hypotheses of more efficient processing after training (Hempel et al., 2004; Sayala et al., 2006; Belleville et al., 2014). However, the BOLD changes observed after Cogmed training do not include only deactivations. Subcortical increases in activity were found By Brehmer et al. in areas restricted to the thalamus and a middle frontal region (WM-low) and caudate (WM-high; Brehmer et al., 2011). We also observed that the Cogmed group showed an increased hemodynamic response in the parietal lobe during the WM-low load condition compared to the WM-high load. Additionally, the Cogmed group displayed enhanced activation in the occipital lobe during the WM-high load task, compared to the WM-low load task. Emery et al. also find training-related increases in the activation of WM network brain areas, such as the bilateral prefrontal cortex, in older adults in relation to younger adults (Emery et al., 2008). These findings suggest potential age and processing demands-differences in the activation of the WM network evoked by adaptive training. In any case, brain function plasticity can be found in adults in response to training with repeated and adaptive WM tasks, as seen in other studies (Hempel et al., 2004; Emery et al., 2008; Brehmer et al., 2011; Vermeij et al., 2017).

Although neural activity in regions recruited by WM tasks was found, the groups did not differ for the in-scanner WM tasks, which is in line with other studies that found no behavioral changes during the fMRI assessment (Hempel et al., 2004; Sayala et al., 2006; Brehmer et al., 2011). This may be probably because the neural and behavioral changes associated with repeating WM tasks are not evoked similarly (Sayala et al., 2006).

Despite not observing a significant difference in working memory (WM) performance following Cogmed training, among individuals that made a minimum of 20 sessions of Cogmed training, we found a high correlation between an increased score in the WM composite Z-score and decreased activation in the right superior parietal lobe, a component region of the WM processing networking. This result may indicate that training with Cogmed increased processing efficiency in this region involved in WM, and this was associated with cognitive performance assessed outside the scanner. However, further investigations will be important to understand these functional changes’ maintenance and clinical benefits in large populations.

Lastly, some limitations of the study need to be considered. The small sample size could result in low power to detect small and medium effects, as Cohens’d effect size analysis suggested. These issues need to be improved in future studies with larger samples. In addition, as we finished our study during the Covid-19 pandemic, some patients (*n* = 8) had to do the cognitive evaluation online to minimize contagion risks. However, to ensure the cognitive function evaluation’s accuracy and prevent family members’ interference, we asked the participants to find a peaceful and private place to do the tests. In addition, the video cameras were kept open during the evaluation, and all activities were recorded when they were completed. Although the randomization was performed properly, the control group had a higher frequency of never-smokers than Cogmed, which could bias the analysis once smoking is a risk factor for cognitive impairment. However, the cognitive performance of the Cogmed group was not worsening compared to the control in the post-training analysis. Furthermore, some of the tests used in this study had no different versions available for baseline and post-training evaluations in order to minimize learning and re-test effect, as we did for visuospatial abilities and verbal memory. This limitation could be improved in further studies. Our study also has strengths. As far as we know, this was the first study evaluating the Cogmed effect on hypertensive individuals. Furthermore, we investigate the effect of the training not only in cognition but also in the modulation of neuronal activation indirectly measured by the BOLD signal, which provides reliable evidence from the functional changes. Finally, although Cogmed was not associated with increased WM performance, no decline was seen either in this period. Furthermore, more efficient processing was found in brain areas underlying WM after Cogmed training. Future studies should consider evaluating the long-term clinical benefits of these functional changes.

## Conclusion

No differences were found between the two groups for WM performance or any other cognitive outcome. However, after training, the Cogmed group had a decreased activation in the right superior parietal lobe and left anterior frontal lobe. In addition, among the Cogmed participants that made at least 20 sessions of Cogmed training, a higher composite Z-score of WM was correlated with lower beta values of activation in the right superior parietal lobe obtained during the in-scanner WM-low load task. These results suggest a post-training increase in neuronal efficiency in areas underlying WM.

## Data availability statement

The raw data supporting the conclusions of this article will be made available by the authors, without undue reservation.

## Ethics statement

The studies involving human participants were reviewed and approved by Comissão de Ética para Análise de Projetos de Pesquisa do HCFMUSP. The patients/participants provided their written informed consent to participate in this study.

## Author contributions

RP: study concept and design, data acquisition, analysis, interpretations of data, and manuscript writing. BC: data acquisition, analysis, and critical revision of the manuscript for intellectual content. MN: data acquisition and critical revision of the manuscript for intellectual content. NF, FC-C, LB, and DC: critical revision of the manuscript for intellectual content. LT and LM: cognitive evaluation. MI: study concept and design, interpretations of data, critical revision of the manuscript for intellectual content, and study supervision. All authors contributed to the article and approved the submitted version.

## Funding

This work was supported by CAPES (grant number 88882.179915/2018-01), FAPESP (grant number 2018/19006-2), and CNPq (grant number 307138/2015-1).

## Conflict of interest

The authors declare that the research was conducted in the absence of any commercial or financial relationships that could be construed as a potential conflict of interest.

## Publisher’s note

All claims expressed in this article are solely those of the authors and do not necessarily represent those of their affiliated organizations, or those of the publisher, the editors and the reviewers. Any product that may be evaluated in this article, or claim that may be made by its manufacturer, is not guaranteed or endorsed by the publisher.

## Footnotes

^1^www.fmrib.ox.ac.uk/fsl/
