# Supplementary material for: Computerized working memory training for hypertensive individuals with executive function impairment: a randomized clinical trial
Source: Front Neurosci. 2023 Jul 7;17:1185768. doi: 10.3389/fnins.2023.1185768 (PMC10361818; doi:10.3389/fnins.2023.1185768)
Supplement: Supplementary file 1 [file Data_Sheet_1.docx]

**Computerized Working Memory Training for Hypertensive Individuals With Executive Function Impairment: a Randomized Clinical Trial**

Regina Silva Paradela^a^; Brenno Cabella^b^; Mariana Penteado Nucci^c^; Naomi Vidal Ferreira^d^; Laura Aló Torres^a^; Luiza Menoni Martino^a^; Fernanda Marciano Consolim-Colombo^a^; Luiz Aparecido Bortolotto^a^; Danielle Irigoyen da Costa^e^; Maria Claudia Irigoyen^a^

^a^Instituto do Coracao (InCor), Hospital das Clinicas HCFMUSP, Faculdade de Medicina, Universidade de Sao Paulo, Sao Paulo, SP, BR.

^b^Institute of Theoretical Physics, Sao Paulo State University (IFT-UNESP), Sao Paulo, SP, BR.

^c^Laboratory of Medical Investigations on Magnetic Resonance Imaging (LIM‐44), Hospital das Clinicas HCFMUSP, Faculdade de Medicina, Universidade de Sao Paulo, Sao Paulo, SP, BR.

^d^University of Sao Paulo Medical School, São Paulo, SP, Brazil/Adventist University of Sao Paulo, Engenheiro Coelho, SP, BR.

^e^Brain Institute (InsCer), Pontifícia Universidade Católica do Rio Grande do Sul (PUCRS), Porto Alegre, RS, BR.

**Running title:** Cognitive training for hypertensive patients

**Keywords:** Executive function; Cognitive Dysfunction; Rehabilitation; Hypertension; Magnetic Resonance Imaging.

**Corresponding author:** Regina S. Paradela, 455 Doutor Arnaldo Avenue, room 1355, São Paulo, SP, Brazil. E-mail: [reginarpds@gmail.com](mailto:reginarpds@gmail.com)

**Supplemental Methods**

*Functional magnetic resonance acquisition parameters*

Blood-oxygen-level-dependent fMRI images of the whole brain were acquired with a gradient-echo-planar-imaging (EPI) pulse sequence according to the following parameters: TR/TE=2500/30 ms, flip angle=90°, matrix=80x80, FOV=240x240 cm, with 42 slices (slice thickness of the 3 mm and 0.3 mm interslice spacing). Slices were acquired interleaved in axial orientation. Brain images were acquired in 3D high-resolution T1 weighted anatomical image with repetition time/ echo time of 7.0 / 3.2 ms, flip angle 8^o^, Sense 1.5, a field of view of 240 × 240, matrix 240 × 240, 180 slices of 1 mm each with no gap, and voxel size of 1 mm³.

**Supplementary Tables**

Supplementary Table 1. Control group training.

| **Games** | **Description** |
| --- | --- |
| Naval battle | Participants had to hit the green target to gain ammo. Red should be avoided. They moved the tank using the "left" and "right" arrow and the target using the "up" and "down" arrows on the keyboard. To shoot, they used the "space" key on the keyboard. |
| Tic-tac-toe | The participants played against the computer. Whoever gets 10 points first wins. An 'X' should be clicked on the desired area. |
| Nim game | The participant and the computer could cross out 1 to 4 toothpicks at a time. In the end, whoever got the bomb lost. The participant could choose the number of toothpicks he wanted to cross out by clicking on the number or typing on the keyboard. |
| Hangman | A gallows appeared on the screen, and the participant clicked any letter on the keyboard until they hit a word or the game ended. |

**Supplementary Table 2**. Correlation of the beta values derived from the post-training activation of working memory (WM)-Low and WM-high contrast maps with the post-training composite Z-score of WM evaluated outside the scanner.

|  | **Composite Z-score of WM** | |
| --- | --- | --- |
| **Cogmed group** | r | P-value |
| *WM-low - ITT analysis (n=15)* |  |  |
| Right superior parietal lobe | -0.42 | 0.12 |
|  |  |  |
| *WM-low - PP analysis (n=11)* |  |  |
| Right superior parietal lobe | -0.75 | 0.007 |
|  |  |  |
| *WM-high - PP analysis (n=11)* |  |  |
| Left anterior frontal lobe | -0.34 | 0.30 |
|  |  |  |
| **Control group** |  |  |
| *WM-low - ITT analysis (n=11)* |  |  |
| Right superior parietal lobe | 0.0002 | 0.99 |
|  |  |  |
| *WM-low - PP analysis (n=9)* |  |  |
| Right superior parietal lobe | 0.12 | 0.76 |
|  |  |  |
| *WM-high - PP analysis (n=9)* |  |  |
| Left anterior frontal lobe | 0.37 | 0.32 |

Values represent Pearson's correlation coefficient r with P-value.

**Supplementary Figures**

**Supplementary Figure 1.** Results for post-training lower-level contrast (Working Memory-Low load > Working Memory-High load conditions) of the Cogmed group. Yellow/red areas indicate significant activation in parietal lobe areas (Cluster size: 409 voxels; brain coordinates of the cluster peak: x = 2, y = -62, and z = 30; z max: 3.51, p<0.05).

**Supplementary Figure 2.** Results for post-training lower-level contrast (Working Memory-High load> Working Memory-Low load conditions) of the Cogmed group. Yellow/red areas indicate significant activation in occipital lobe areas (Cluster size: 509 voxels; brain coordinates of the cluster peak: x = 30, y = -86, and z = 4; z max: 3.37, p<0.05).
