# Supplementary figures and images for: Computerized working memory training for hypertensive individuals with executive function impairment: a randomized clinical trial
Source: Front Neurosci. 2023 Jul 7;17:1185768. doi: 10.3389/fnins.2023.1185768 (PMC10361818; doi:10.3389/fnins.2023.1185768)

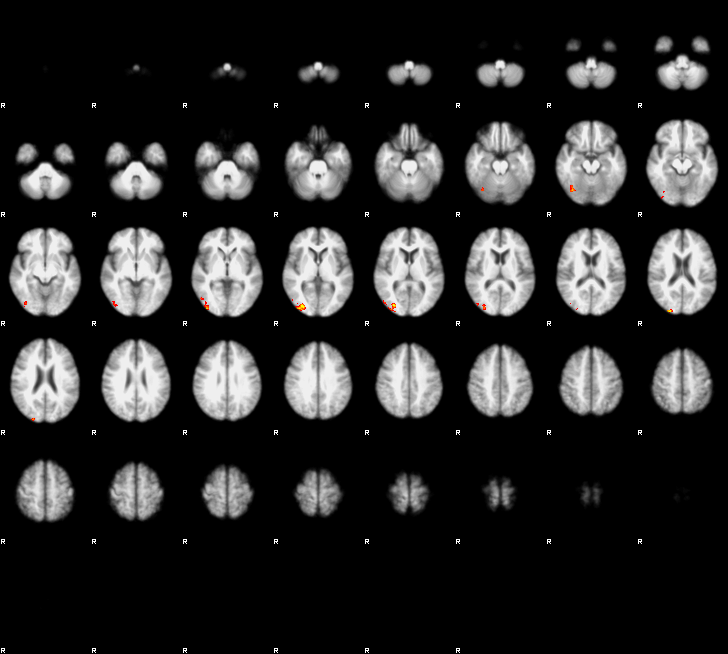

Supplement: Supplementary file 2 [file Image_1.PNG]

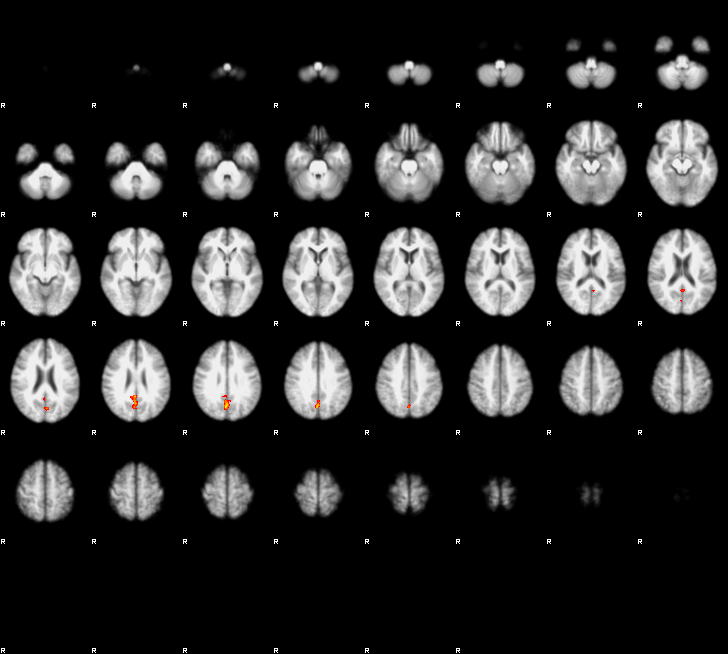

Supplement: Supplementary file 3 [file Image_2.PNG]
